# Luminance noise impacts putative luminance components of visual evoked potentials but not chromatic components

**DOI:** 10.1186/s41155-025-00348-z

**Published:** 2025-07-28

**Authors:** Bellany Barbosa Lopes, Terezinha Medeiros Gonçalves Loureiro, Felipe André da Costa Brito, Letícia Miquilini, Alódia Brasil, Marcelo Fernandes Costa, Railson Cruz Salomão, Dora Fix Ventura, Ana Leda Brino, Givago da Silva Souza

**Affiliations:** 1 Núcleo de Teoria e Pesquisa do Comportamento, Universidade Federal do Pará, Belém, Pará, Brazil; 2 Instituto de Ciências Biológicas, Universidade Federal do Pará, Belém, Pará Brazil; 3 Instituto de Ciências da Saúde, Universidade Federal do Pará, Belém, Pará Brazil; 4 Instituto de Psicologia, Universidade de São Paulo, São Paulo, Brazil; 5 Núcleo de Medicina Tropical, Universidade Federal do Pará, Belém, Pará Brazil

**Keywords:** Visual evoked cortical potential, Pseudoisochromatic stimulus, Color vision, Luminance vision, Visual electrophysiology

## Abstract

**Introduction:**

Pseudoisochromatic stimuli are widely used in psychophysical color vision testing and the features of the luminance noise present on these stimuli have been reported modifying the psychophysical chromatic discrimination.

**Objective:**

The present study investigated how modifications in the luminance noise features (luminance contrast and number of luminance values) affect chromatic visual evoked cortical potentials (VECP) elicited by pseudoisochromatic gratings, aiming to evaluate the influence of luminance contrast and the number of luminance values in the pseudoisochromatic stimulus on the chromatic VECP.

**Methods:**

The sample consisted of seven young trichromatic participants. The waveforms of the visual evoked cortical potentials (VECP) were analyzed, focusing on the P1, N1, and P2 components across all stimulus conditions. The luminance noise contrast in the pseudoisochromatic stimulus had distinct effects on the amplitudes of the VECP components. Significant effects were observed for the amplitudes of the P1 (*p =* 0.01) and P2 (*p =* 0.04) components, while no significant effect was found on the amplitude of the N1 component (*p =* 0.3).

**Results:**

There was no significant effect of the luminance noise range on the latency of the VECP components (P1 VECP component, *p =* 0.54; N1 VECP component, *p =* 0.79; P2 VECP component, *p =* 0.49). The number of luminance values in the noise had no significant effect on VECP components amplitude and latency. Different manipulations of luminance noise influenced P1 and P2 VECP components and no modification of the luminance noise had influence on the main chromatic VECP component, N1 component.

**Conclusion:**

Manipulations in features of the luminance noise in pseudoisocrhomatic stimulus impacted in putative luminance components, but not chromatic components, of visual evoked potentials. The present findings may have potential applications in clinical neuro-ophthalmology, particularly for assessing congenital and acquired color blindness.

## Introduction

Pseudoisochromatic stimuli incorporate luminance noise that eliminates brightness differences between the target stimulus and the background, enabling target detection to rely solely on chromatic cues (Mollon, 2003). The luminance noise in these stimuli consists of a random distribution of luminance values spread across the mosaic, uniformly spaced between a defined minimum and maximum luminance level.

Most studies utilizing pseudoisochromatic stimuli have primarily focused on their clinical application for identifying congenital and acquired color vision deficiencies. However, recent investigations have shown that manipulating luminance noise parameters can provide novel insights into the visual system beyond those traditionally obtained through such tests. For instance, several psychophysical studies have examined how modifications in luminance noise settings influence chromatic discrimination in trichromats and dichromats (Souza et al., 2014; Cormenzana-Mendez et al., 2016; Linhares et al., 2016; Loureiro et al., 2018).

In general, these studies suggest the existence of color-luminance interactions that play a significant role in the perception of pseudoisochromatic stimuli. For example, Loureiro et al. (2018) observed that changes in the minimum and maximum luminance values of the noise significantly affected the color perception of dichromats but not trichromats. Additionally, Souza et al. (2014) reported that increasing the number of luminance levels within the noise led to poorer chromatic discrimination in pseudoisochromatic arrangements.

The mechanisms underlying the effects of luminance noise masking on chromatic discrimination using pseudoisochromatic stimuli remain unclear. Non-invasive electrophysiological tools, such as visual evoked cortical potentials (VECPs), may help elucidate how early cortical mechanisms process luminance-chromatic interactions during the perception of pseudoisochromatic stimuli. VECPs have been widely used to record cortical activity related to luminance and chromatic contrast processing (Kulikowski et al., 2002). Previous studies have demonstrated distinct functional signatures of visual pathways for chromatic and luminance processing (Campbell & Maffei, 1970; Valberg & Rudvin, 1997; Rudvin et al., 2000; Gerth et al., 2003; Crognale et al., 2013; Zemon & Gordon, 2006; Gomes et al., 2006, 2008, 2010; Souza et al., 2007, 2008, 2013; Araújo et al., 2013; Risuenho et al., 2015; Martins et al., 2019).

A frequently used approach to differentiate luminance and chromatic signals involves recording VECPs elicited by pattern-onset stimulation using isoluminant or cone-specific stimuli (Carden et al., 1985; Suttle & Harding, 1999; Porciatti & Sartucii, 1999; Rabin et al., 1994; Crognale et al., 2013; Gerth et al., 2003; Gomes et al., 2006, 2008, 2010; Souza et al., 2008; Rabin et al., 2016). Under pattern-onset stimulation, a luminance contrast stimulus evokes a cortical waveform with a positive component around 100 ms, while an isoluminant chromatic stimulus elicits a waveform dominated by a negative component within the same time window (Carden et al., 1985). This negative component is abolished in colorblind individuals or diminished when the chromatic stimulus contains luminance contrast artifacts (Kulikowski et al., 1996; Suttle & Harding, 1999; Gomes et al., 2006). Notably, chromatic discrimination thresholds estimated from chromatic pattern-onset VECPs have been correlated with psychophysical chromatic discrimination (Gomes et al., 2006, 2008).

Salomão et al. (2019) introduced the use of pseudoisochromatic stimuli as an alternative to evoke cortical responses to chromatic stimuli. This approach has the advantage of not requiring psychophysical photometric procedures to establish isoluminance between the chromatic components of the stimulus. The resulting waveforms elicited by pattern-onset pseudoisochromatic gratings were comparable to those generated by isoluminant chromatic gratings under pattern-onset conditions.

Our hypothesis is that the VECPs elicited by pseudoisochromatic gratings reflect chromatic mechanisms associated with psychophysical chromatic discrimination. Moreover, we propose that the chromatic components of the pattern-onset VECPs should be influenced by modifications in the luminance noise configuration. Conversely, the absence of luminance noise effects on chromatic cortical responses would provide critical insights into the functional substrates of these responses.

The present study aimed to evaluate how changes in the luminance noise configuration of pseudoisochromatic stimuli affect chromatic pattern-onset VECPs. By exploring this, we aim to deepen our understanding of the cortical mechanisms underlying luminance-chromatic interactions.

## Methods

### Ethics

All procedures in the present study were approved by the by the Ethical Committee for Research in Human of the Federal University of Pará, Brazil (report 991.803). All participants gave a written informed consent to participate in the experiments.

### Subjects

Seven healthy subjects participated in the experiments (3 males, 4 females, 26.5 ± 3.4 years-old). All had normal visual acuity or optically corrected to 20/20 or better, and were normal trichromats. The color vision phenotype was diagnosed using the Cambridge Colour Test (CCT) (Cambridge Research System (CRS), Rochester, United Kingdom). None had history of neurological or systemic diseases that affected the normal function of the visual system.

### Stimulus

The stimulus was generated in the ViSaGe System (Cambridge Research System, CRS, Rochester, UK) using a MATLAB environment (R2017b, Mathworks, Natick, MA) and CRS toolbox for MATLAB (CRS). It was presented in a 21’’ color CRT monitor (Model Mitsubishi Diamond PRO 2070, Mitsubishi Electric Australia, Rydalmere, Australia) with high spatial and temporal resolution (1280 x 1024 pixels, 75 Hz). Before the experiments the visual display was gamma-corrected using ColorCal II chromameter (CRS) and the software vsgDesktop (CRS).

**Fig. 1:**
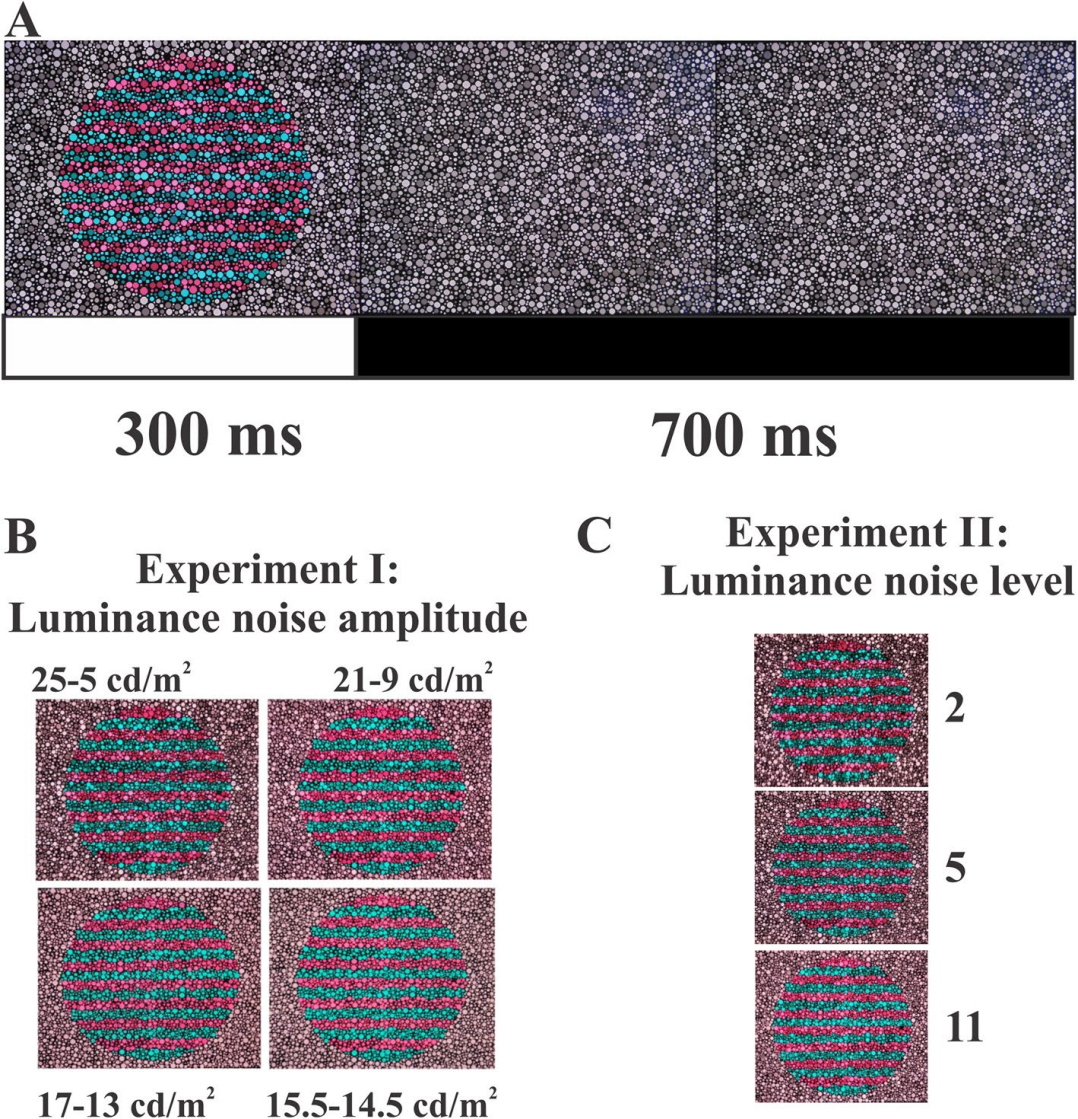
Stimuli examples: **A** shows presentation; **B** four conditions for Experiment I; **C** three conditions for Experiment II

The stimulus was a 7.25° field containing a 6° circular patch with pseudoisochromatic 2 cpd red-green horizontal grating (Fig. 1). This spatial frequency was used to maximize the VECP amplitude as previously observed observed (Porciatti & Sartucii, 1999; Salomão et al., 2019). The entire field was composed of circular patches of varying diameter, whose luminance spread over four different ranges in Experiment I (see Table 1) and three different levels in Experiment II (see Table 2).

**Table 1:** Experimental conditions of the Experiment I. The conditions differed in the range of luminance in the noise, but they had the same mean luminance and number of luminance values in the noise. The range is the difference between maximum and minimum luminance values

Luminance noise amplitude	Max luminance	Min luminance
20 cd/m2	25 cd/m2	5 cd/m2
12 cd/m2	21 cd/m2	9 cd/m2
4 cd/m2	17 cd/m2	13 cd/m2
1 cd/m2	15.5 cd/m2	14.5 cd/m2

*Max* maximum, *Min* minimum

**Table 2:** Experimental conditions of the Experiment 2. The conditions differed in the number of luminance values in the noise, but they had the same mean luminance and the same range of luminance in the noise

**Number of luminance values in the noise**	**Luminance values in the noise (cd/m**^**2**^**)**
2 values	25 and 5
5 values	25, 20, 15, 10, and 5
11 values	25, 23, 21, 19, 17, 15, 13, 11, 9, 7, and 5

The chromatic gratings were red (CIE1976 coordinate, u’= 0.267, v’= 0.469) and green (CIE1976 coordinate, u’= 0.1287, v’= 0.469 and the background was an achromatic point in the chromaticity diagram (CIE1976 coordinate, u’= 0.1977, v’= 0.469). The stimulus pattern was shown during 300 ms along with the background (onset period), and during the following 700 ms only the background was shown (offset period).

The present study was composed by two experiments that modified the luminance noise configuration of pseudoisochromatic gratings that were used to elicit VECPs. The Experiment I investigated how the range of luminance in the noise of pseudoisochromatic stimulus influenced on the chromatic pattern-onset VECP. We used four different ranges of luminance in the noise keeping the same mean luminance and the number of luminance values equally spaced inside the range of luminance of the noise as observed in the Table 1.

The Experiment II to investigate how the number of luminance values in the noise of pseudoisochromatic stimulus influences on the chromatic VECP. Three luminance noise configurations having 2, 5, and 11 luminance values equally apart from the minimum and maximum luminance values were tested. Again, all conditions share the same mean luminance and have the same range of luminance as observed in the Table 2.

### Recordings

Subjects were tested binocularly, and all had normal or best-corrected visual acuity to 20/20. One gold-cup surface electrodes channel was placed on the scalp over the Oz (active electrode), Fpz (reference electrode), and Fz (ground electrode) following the International Society for Clinical Electrophysiology of Vision (Odom et al., 2016). Electrode impedance was maintained below 5 kΩ. The electroencephalographic potentials were amplified 30,000 times by a differential amplifier (CED 1902 model, Cambridge Electronic Design, CED, Cambridge, United Kingdom). The signal thus obtained was digitized at a rate of 1000 Hz (CED). Spike 2 software (CED) was used to drive the recordings and save them in text files in the computer. The recording was triggered by the stimulus through a TTL signal sent from the ViSaGe system to the CED system. For each stimulus condition, we recorded 240 sweeps of 1 s duration. The total time of testing was about 30 min.

### Statistics

For all stimulus conditions, the amplitude and latency from three VECP components (P1, N1, and P2) were measured. The VECP amplitudes were the voltage difference between the baseline (mean amplitude between 0 and 10 ms) and the VECP component peak (Gomes et al., 2006, 2008; Souza et al., 2008; Salomão et al., 2019) the VECP latency was the time difference between the stimulus onset and the VECP component peak. We used Friedman tests followed by Dunn’s multiple comparison test to evaluate the effect of the different ranges of luminance in the noise and the number of luminance values in the noise on VECP components amplitude and latency and considered level of significance equal to 0.05.

## Results

### Influence of the range of luminance in the noise on pseudoisochromatic pattern-onset VECP components

**Fig. 2:**
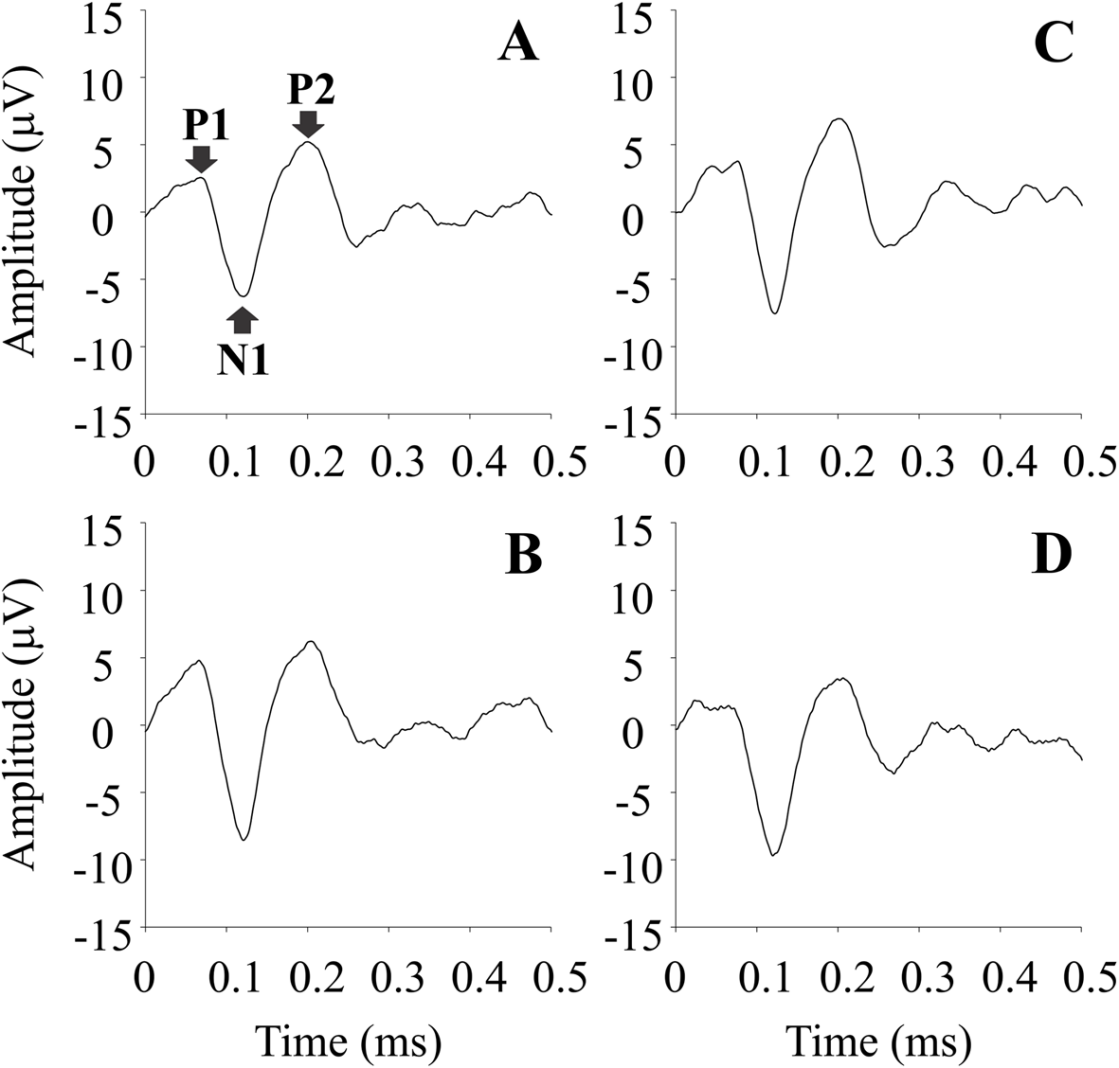
Médias das gravações para todas as condições do Experimento I, mostrando formas de onda e amplitudes de P1, N1 e P2 para cada ruído de luminância

For all stimulus conditions, we found waveforms composed by P1, N1, and P2 components. The grand-mean waveforms elicited by each condition of range of luminance in the noise are shown in the Fig. 2.

Median and interquartile range of the VECP components amplitude and latency are shown in Table 3. We found that the conditions of range of luminance in the noise had different effects on the VECP component amplitudes. Significant effects were observed for P1 and P2 VECP component amplitudes (P1 VECP component amplitude: Friedman statistic = 10.48, *p =* 0.01; P2 VECP component amplitude: Friedman statistic = 7.97, *p =* 0.04), while no significant effect was observed in the N1 VECP component amplitude (N1 VECP component amplitude: Friedman statistic = 3.618, *p =* 0.3). The stimulus condition of luminance noise ranging between 15.5 and 14.5 cd/m^2^ elicited P1 VECP component with smaller amplitude than the stimulus condition with luminance noise ranging between 17 and 13 cd/m^2^ and between 21 and 9 cd/m^2^. The P2 VECP component amplitude was smaller in the luminance noise ranging from 15.5 to 14.5 cd/m^2^ than in the luminance noise condition ranging between 25 and 5 cd/m^2^.

**Table 3:** VECP components median (interquartile range) amplitudes and latencies for the different ranges of luminance in the noise of the pseudoisochromatic stimulus

	**P1**		**N1**		**P2**	
**Luminance noise amplitude**	**Amplitude (**μ**V)**	**Latency (ms)**	**Amplitude (**μ**V)**	**Latency (ms)**	**Amplitude (μV)**	**Latency (ms)**
20 cd/m^2^	3.29(1.2)	62(35)	8.63(4.9)	116(8.5)	5.32(2.5)	192(24)
12 cd/m^2^	4.27(1.2)	72(16)	8.58(7.4)	119(23.5)	5.34(8)	195(20)
4 cd/m^2^	5.03(2.2)	60(14.5)	8.6(8.3)	118(9)	7.63(5.9)	174(52)
1 cd/m^2^	2.11(4.1)*	64(16.5)	10.51(1.6)	115(9.5)	3.45(3.2)**	193(19)

^*^Adjusted *p*-value for multiple comparison < 0.05 compared to 12 and 4 cd/m^2^ conditions of luminance noise amplitude

^**^Adjusted *p*-value for multiple comparison < 0.05 compared to 20 condition of luminance noise amplitude

There was no significant effect of the different ranges of luminance in the noise on the latency of the VECP components (P1 VECP component latency: Friedman statistic = 2.162, *p =* 0.54; N1 VECP component latency: Friedman statistic = 1.062, *p =* 0.79; P2 VECP component latency: Friedman statistic = 2.426, *p =* 0.49).

### Influence of the number of luminance values in the noise on pseudoisochromatic pattern-onset VECP components

**Fig. 3:**
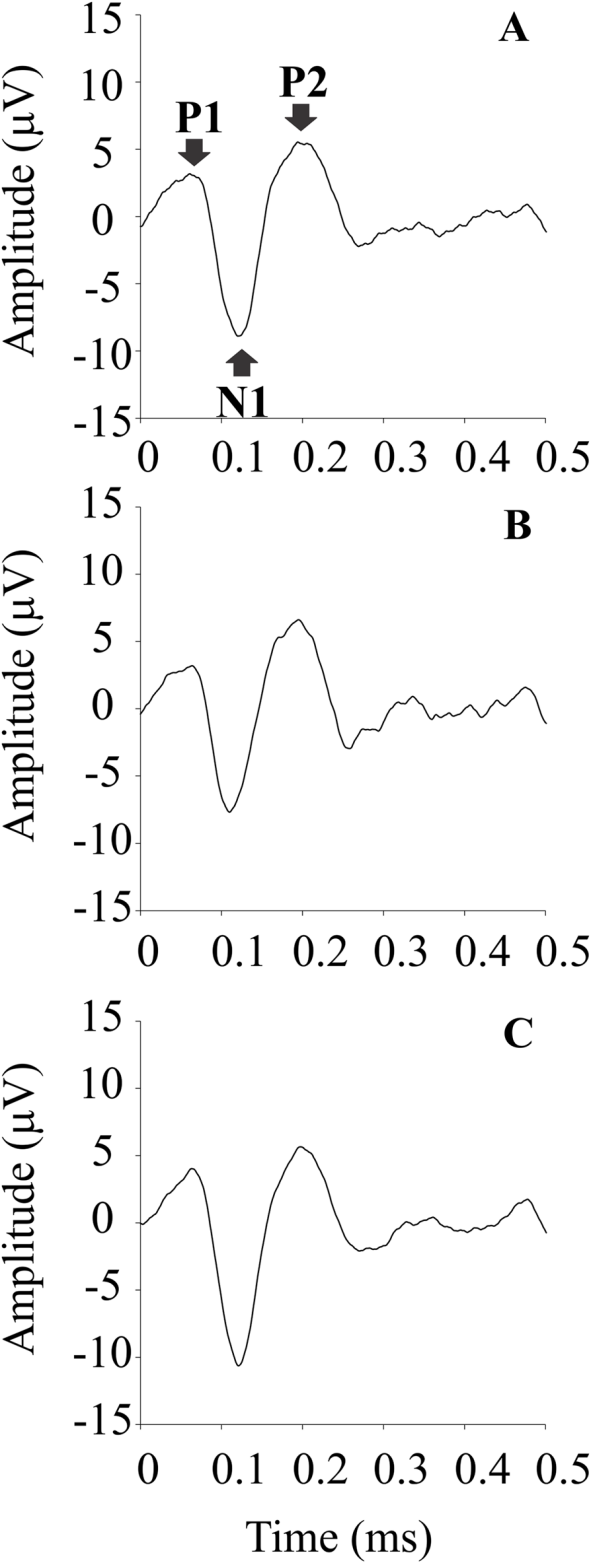
Mean recordings for Experiment II conditions showing waveforms and P1, N1, P2 amplitudes over time

In this experiment, we also found waveforms contained P1, N1, and P2 components. The grand-mean waveforms elicited by each number of luminance values in the noise are shown in Fig. 3.

The number of luminance values in the noise had no significant effect on VECP components amplitude (P1 VECP component amplitude: Friedman statistic = 0.2857, *p =* 0.964; N1 VECP component amplitude: Friedman statistic = 0.2857, *p =* 0.964; P2 VECP component amplitude: Friedman statistic = 1.143, *p =* 0.62), and latency (P1 VECP component latency: Friedman statistic = 0.8571, *p =* 0.7682; N1 VECP component latency: Friedman statistic = 2.385, *p =* 0.3214; P2 VECP component latency: Friedman statistic = 2.296, *p =* 0.3581).

Table 4 shows the median and interquartile range of the VECP components amplitude and latency for each condition of number of luminance values in the noise.

**Table 4:** VECP components median (interquartile range) amplitudes and latencies for the different conditions of amount of luminance values in the noise of the pseudoisochromatic stimulus

	**P1**		**N1**		**P2**	
**Luminance noise level**	**Amplitude (μV)**	**Latency (ms)**	**Amplitude (μV)**	**Latency (ms)**	**Amplitude (μV)**	**Latency (ms)**
2 levels	3.33(2.2)	64(21.5)	8.63(9.3)	125(11)	4.92(7.3)	205(14)
5 levels	3.75(2.9)	47(24)	9.9(9.1)	115(13.5)	5.32(8.4)	180(34.5)
11 levels	5.07(4)	63(3.5)	8.97(10.1)	117(4.5)	3.79(6.4)	193(14)

## Discussion

Salomão et al. (2019) introduced the VECP elicited by pseudoisochromatic gratings. They observed that the waveforms of chromatic VECP had similar components to those elicited by isoluminant chromatic sinusoidal gratings and these components were decreased or absent in dichromatic subjects. The present study aimed to extend the knowledge about pseudoisochromatic pattern-onset VECPs testing the influence of different configuration of the luminance noise on the cortical response. Similar changes in the luminance noise we tested in the present study had significant influence on perceptual chromatic discrimination in previous psychophysical experiments (Souza et al., 2014; Loureiro et al., 2018). Our main finding was that different manipulations of the luminance noise had different influence on chromatic pattern-onset VECP components. Only changes in the range of luminance in the noise had significant influence on the P1 and P2 VECP components. No luminance noise modification had significant influence on the N1 chromatic pattern-onset VECP component.

Our interpretation for the results is that the modifications in the luminance noise, mainly the changes of the range of luminance in the noise, impacted in the global luminance contrast of the stimulus, from high- to low-contrasts. These changes had some influence in the positive chromatic pattern-onset VECP (P1 and P2 components). P1 and P2 components had smaller amplitude in conditions of low luminance contrast. The changes of the number of luminance values in the noise led to changes in noise contrast, from high to intermediate contrast, but the results showed that these contrast changes were not enough to observe significant decrease of VECP components.

The contribution of color-opponent pathways to the positive onset chromatic VECP (P1 and P2 components) is largely unknown. The P1 onset VECP component is frequently absent or has small amplitude when it is elicited by isoluminant chromatic gratings, and presents maximal amplitude for achromatic sinusoidal gratings (Porciatti & Sartucci, 1999; Souza et al., 2008). In the present data, the smallest amplitude of P1 pattern-onset VECP component occurred in the luminance noise condition with the smallest range of luminance (low-contrast), i.e., when the stimulus was close to the luminance homogeneity, suggesting the detection of some global luminance information from the luminance noise. The P2 pattern-onset chromatic VECP component is usually present in the chromatic pattern-onset VECP, but no systematic investigation has been previously reported. Porciatti and Sartucci (1999) observed that the P2 chromatic pattern- onset VECP component elicited by chromatic gratings with different color ratios has a smaller amplitude in the isoluminant condition than in the conditions with overlapping luminance and color. Here, we observed smaller P2 VECP component amplitudes also occurred in the noise condition with luminance ranging 15.5 and 14.5 cd/m^2^. Our findings and previous observations suggest that the P2 VECP component could be a discrete physiological substrate of the beginning of the cortical luminance-color interaction or both the differences in these positive components can represent only luminance artifacts.

N1 is the most studied component among the chromatic pattern-onset VECP components (Carden et al., 1985; Rabin et al., 1996; Porciatti & Sartucci, 1999; Gomes et al., 2006; Souza et al., 2008). It has been reported as a good predictor of chromatic iscrimination (Carden et al., 1985; Gomes et al., 2006; Souza et al., 2008), because its amplitude decreases as the luminance contrast is added to the chromatic isoluminant stimulus, its polarity is inverted for the onset of achromatic stimulus, the interpolation of its amplitude as a function of the chromatic difference in the stimulus predicts the psychophysical chromatic discrimination threshold, and it is absent in congenital dichromacy (Gomes et al., 2006). In the present study, we observed that the N1 chromatic pattern-onset VECP component had no significant change across the multiple stimulus conditions tested. We interpreted that this component reflects chromatic cortical mechanisms that are not involved in luminance-color cortical interactions observed in psychophysical and electrophysiological experiments (Souza et al., 2014; Xing et al., 2015; Comenzana-Mendez et al., 2016; Miquilini et al., 2017; Loureiro et al., 2018; Sousa et al., 2020; Brito et al., 2022).

Although the present study used a sample consisting of only seven participants, the reliability of the data obtained should not be disregarded. Studies investigating visual evoked potentials (VEPs) often rely on small samples due to the controlled nature and methodological rigor required to record precise neurophysiological responses, which contributes to the relevance of the analyses (Maxwell, 2004). According to Marmoy et al. (2024), research on chromatic VEPs has been expanding, although, due to the complexity of experimental methods, it still depends on small samples. The reliability of data in small samples is reinforced by the consistency of measurements and the replicability of the methodologies used. Moreover, the differences observed between experimental conditions were statistically significant, indicating that the experimental manipulations were appropriate for recording accurate responses. For future investigations, larger samples may expand the generalization of the results.

One alternative explanation for the observed effects of luminance noise on the chromatic pattern onset VECP suggests that these effects may be related to visual attention processes. Luminance noise can create areas in the visual field with similar luminances, acting as distractions that compete for attention with the chromatic stimulus. This competition for attention could result in a shift in visual focus, redirecting it towards luminance, which would negatively impact psychophysical performance in the chromatic discrimination task. However, this attentional shift would not directly alter the VECPs, as these potentials represent a neural response to the stimuli, regardless of how attention is allocated.

Although attentional mechanisms have been extensively described in the primary visual cortex—the first brain region to process visual stimuli—the complexity of these mechanisms tends to increase as processing extends to extrastriate visual areas (Motter, 1993; Roelfsema et al., 1998). In the extrastriate cortex, visual attention engages regions responsible for processing more complex attributes, such as shape, motion, and color perception, further influencing how visual information is prioritized and interpreted. Consequently, visual attention not only enhances focus on specific stimuli but also modulates the neural representation of sensory information. Eye-tracking or attention measurement tasks could help quantify the impact of attention on the luminance and chromatic waveforms we recorded using pseudoisochromatic gratings.

The present study focused on the luminance modulation in pattern-onset chromatic gratings, because the waveform is very stable and known about its physiological meaning, but to explore more aspects of the color-luminance interactions of the cortical visual processing, future studies can incorporate more complex patterns such as checkboard or concentrical rings or dynamic modulation of the noise features.

It is well known that cortical pathways for color and luminance can serve as neurological markers for congenital and acquired losses in visual processing mechanisms. Exposure to chemicals and chronic degenerative diseases such as diabetes and multiple sclerosis are examples of conditions that can lead to acquired impairments in color and luminance discrimination (Lacerda et al., 2012; Andrade et al., 2014; Lampert et al., 2015; Lacerda et al., 2020). Investigating the cortical response to the activation of each of these mechanisms could be useful for the early identification of neural damage or even for disease staging. Similarly, congenital conditions such as dichromacy or cerebral achromatopsia (Barboni et al., 2019; Martins et al., 2019) may have their diagnosis supported by recording the cortical responses examined in the present study. Since visually evoked cortical potentials require minimal cooperation from the participant, this tool may be ideal for certain populations with limited ability to collaborate.

From a practical standpoint, our findings could enhance the investigation of congenital and acquired color vision deficiencies in neuro-ophthalmological clinics, while also providing a novel approach to evaluating the cortical processing physiology of chromatic and luminance mechanisms, as well as their interactions.

## Conclusion

The present investigation confirms the pseudoisochromatic stimulus as an important alternative stimulus to elicit chromatic pattern-onset VECPs. The main interpretation for the present results is that the luminance noise information was not essential to isolate color-specific cortical chromatic mechanisms and could be applied in non-invasive electrophysiological evaluation of the color vision with no concerns about the configuration of its luminance noise.

## Data Availability

Data will be made available on request.

## Abbreviations

CCT: Cambridge Colour Test
CIE: International Commission on Illumination
CED: Cambridge Electronic Design
CRS: Cambridge Research System
Fpz: Reference electrode
Fz: Ground electrod
Max: Maximum
Min: Minimum
Oz: Active electrode
VECP: Visual evoked cortical potentials
